# Angiopoietin-1 treated early endothelial outgrowth cells (eEOCs) are activated in vitro and reduce renal damage in murine acute ischemic kidney injury (iAKI)

**DOI:** 10.1186/1471-2369-14-227

**Published:** 2013-10-21

**Authors:** Daniel Patschan, Jörg Rinneburger, Nazif Idrizi, Rico Backhaus, Katrin Schwarze, Elvira Henze, Susann Patschan, Gerhard A Müller

**Affiliations:** 1 Department of Nephrology and Rheumatology, University Hospital of Göttingen, Robert-Koch-Straße 40, 37077 Göttingen, Germany

**Keywords:** Angiopoietin-1, eEOCs, AKI

## Abstract

**Background:**

Acute kidney injury (AKI) severely worsens prognosis of hospitalized patients. Early Endothelial Outgrowth Cells act protective in murine acute ischemic renal failure and renoprotective actions of eEOCs have been documented to increase after cell pretreatment with 8-O-cAMP and Melatonin. Angiopoietin-1 is critically involved in maintaining vascular integrity and regeneration. Aim of the study was to analyze the consequences of eEOC treatment with Ang-1 in murine AKI.

**Methods:**

After 40 minutes of unilateral renal artery clamping with contralateral nephrectomy, male C57/Bl6N mice were injected with either untreated or pretreated (Ang-1) syngeneic murine eEOCs. Two days later serum creatinine levels and morphology were evaluated. Cultured, Ang-1 treated murine eEOCs were analyzed for production/release of proangiogenic and proinflammatory mediators, migratory activity, and cell survival, respectively.

**Results:**

Angiopoietin-1 pretreatment of eEOCs significantly reduced serum creatinine in cell-injected mice. In vitro analysis showed increased migration of Ang-1 treated eEOCs and supernatant from Ang-1 treated eEOCs stimulated migration of cultured mature endothelial cells. In addition, Ang-1 reduced percentages of Annexin V^+^/PI^+^ eEOCs. Intrarenal numbers of eEOCs remained unaffected by Ang-1 and eEOCs did not produce more or less proangiogenic/proinflammatory mediators after being stimulated with Ang-1.

**Conclusions:**

Angiopoietin-1 pretreatment of eEOCs increases the cells’ renoprotective competence in ischemic AKI. Thus, the armentarium of eEOC agonists in AKI is increasingly being expanded and the treatment of AKI with eEOCs becomes a promising future option.

## Background

Acute renal failure (ARF) severely worsens prognosis of hospitalized patients [1]. Approximately 1-5% of all patients treated in the hospital develop ARF during the course of the disease [2,3]. The most common cause is prolonged hypoperfusion (acute ischemic kidney injury – iAKI) [4]. A new therapeutic approach of iAKI was identified in 2006. Goligorsky’s group showed that mice can be protected from iAKI by systemically being injected with endothelial progenitor cells (EPCs) [5]. EPCs have been described for the first time in 1997 [6]. Since then, numerous studies evaluated the role of these cells in vascular diseases [7-12]. Thus, it has widely been accepted that the cells can serve as therapeutic tool in ischemic diseases such as ischemic heart, cerebrovascular, and renal disease [5,13-15].

Since eEOCs (certain subpopulation of EPCs) have been shown to protect the kidney from acute ischemic injury, our major interest focused on strategies to increase the cells’ renoprotective capacity in iAKI.

Angiopoietins are proteins with essential functions in vascular homeostasis [16]. Targeted gene inactivation studies showed that the early stages of vascular development require VEGF whereas Angiopoietin-1 mediates vascular remodeling during later stages [17]. Angiopoietin-2 in contrast can disrupt in vivo angiogenesis [18]. Both proteins competetively interact with the endothelial-specific receptor tyrosine kinase Tie-2 [19]. One fundamental biological role of Angiopoietin-1 is to stabilize interendothelial cell contacts. This results from Angiopoietin-1 induced dephosphorylation of PECAM-1 and VE-Cadherin, respectively [20]. Endothelial cell migration, proliferation, and differentiation on the other hand are also stimulated by the protein [21]. Aim of the study was therefore to investigate the effects of Angiopoietin-1 in the setting of an eEOC-based therapy of iAKI.

## Methods

### Animal models

The animal study protocol was in accordance with the guidelines of the German Institute of Health Guide for the Care and Use of Laboratory Animals and approved by the Institutional Animal Care and Use Committee. C57BL/6 N mice were obtained from Jackson Labs (Bar Harbor, ME, USA) and bred in the local animal facility of the Göttingen University Hospital. All experiments were performed with male 8–12 weeks old C57Bl/6 N mice. All animals were caged separately with a 12:12-h light–dark cycle and had free access to water and chow throughout the study.

### Surgical procedures

Mice were anesthetized (300 μl of 6 mg/100 g ketamine hydrochloride plus 0.77 mg/100 g xylazine hydrochloride) and placed on a heated surgical pad. Rectal temperature was 37°C. After a 1.5-cm midlaparotomy, the left kidney was exposed and clamping of the renal pedicle was performed with microserrefines (Fine Science Tools, Forster City, USA). Second, contralateral nephrectomy was performed in order to induce ischemic AKI. The suture placed around the right renale pedicle was held open until cell injection. A certain volume of eEOC-containing EBM-2 media (0.5 × 10^6^ cells in 50 μl) was injected into the right renal vein (systemic circulation). Very shortly after cell injection, the suture was closed in order to prevent bleeding. The kidney was removed afterwards. Thus, the right kidney was removed before the contralateral clamps were released. Clamping was performed for 40 minutes in all experiments. The vascular clamp was removed about a minute before injecting eEOCs into the right renal vein. The abdominal incision was closed with a 4–0 suture and surgical staples. Two days (48 hours) after this procedure, animals were sacrificed and blood and kidney were collected for further analysis. In each experimental group 8–10 animals were analyzed. Previous own studies showed that creatinine levels peak at 48 hours after unilateral IRI post uni-nephrectomy [5].

### Culture of mouse-derived early endothelial outgrowth cells (eEOCs)

In order to perform cell injection experiments eEOCs were isolated from C57Bl/6 N mice. Therefore, mouse mononuclear cells (MNCs) were enriched by density gradient centrifugation using Biocoll solution (Biochrom, Berlin, Germany) from peripheral blood and spleen cell extracts. The reason for pooling MNCs was to maximize the total number of cells available for injection. Immediately following isolation, mononuclear cells were mixed and 4 × 10^6^ cells were plated in individual wells of a 24-well culture dish, coated with human fibronectin (Sigma, St Louis, MO) and maintained in endothelial cell medium-2 (EGM-2 - Clonetics, Lonza, Walkersville, MD, USA) supplemented with endothelial growth medium (EGM) Single-Quots containing 5% FCS. After 4–5 days of culture, eEOCs were identified by the uptake of DiI-labeled acetylated low density lipoprotein (acLDL) (Invitrogen, Carlsbad, CA, USA) and binding of FITC-labeled BS-1 lectin (BS-1) (Sigma Diagnostics, St. Louis, MO). For this purpose, cells were first incubated with 10 μg/ml DiI-ac-LDL at 37°C for 1 h and later fixed with 2% formaldehyde for 10 min, followed by incubation with BS-1 lectin at 37°C for 1 h. Cells that demonstrated double-positive immunofluorescence in laser scanning microscopy were defined as eEOCs. Laser scanning microscopy was performed using an inverted fluorescence microscope IX-71 (Olympus Deutschland GmbH, Hamburg, Germany) equipped with the appropriate excitation and emission filters (AHF Analysentechnik, Tuebingen, Germany). Images of respective fluorescence channels were recorded as single high resolution 16bit b/w images using a F-View II ext. Camera (Olympus Deutschland GmbH, Hamburg, Germany). The images from every fluorescence channel were then automatically merged using the MFIP-module of the CELL-F® software. In some experiments cells that were used for injection experiments were not labelled with acLDL and BS-1 lectin, but were incubated with CellTracker® (Molecular Probes, Eugene, OR, USA) according to the manufacturer’s protocol.

### In vitro treatment of eEOCs before therapeutic administration

Early Endothelial Outgrowth Cells employed for systemic injections were detached by trypsinization after the first passage, and, after neutralization of trypsin, incubated with CellTracker®. After washing the cells once with PBS, they were resuspended in 50 μl of EGM-2 for systemic injection or for further *in vitro* treatment. For *in vitro* treatment eEOCs were incubated with Ang-1 (250 ng/ml in EGM-2) (Celprogen stem cell research and therapeutics, San Pedro, CA, USA) for 60 minutes at 37°C. After washing the cells once with EGM-2, they were resuspended in 50 μl of EGM-2 for systemic injection.

### Immunofluorescence microscopy

Tissue samples were fixed in a 4% formaldehyde solution for one hour, followed by incubation in 30% sucrose overnight at 4°C. Embedding was performed in an OCT compound (Tissue-Tek, Torrance, CA, USA), and embedded samples were stored at -20°C. Frozen samples were cut into 10 μm thick sections. Non-specific protein binding was blocked by 1 hour incubation with PBS-BSA (1%). Sections were incubated with FITC-conjugated anti-mouse CD117 (c-Kit, 1:1000 in PBS-BSA 1%) (BD Biosciences, Rockville, MD, USA) or with the respective isotype control for 12 hours at 4°C. To visualize the nuclei, tissue sections were counterstained with DAPI (1:200 in PBS) (Molecular Probes, Eugene, OR, USA). Sections were examined as previously described.

### Serum creatinine analysis

Serum creatinine concentration was measured using a commercially available kit (Creatinin PAP, Labor und Technik - Eberhard Lehmann, Berlin, Germany) according to the manufacturer’s protocol.

### Cell migration assays

The eEOC cell migration assay was performed as published by Shi et al. [22]. Briefly, cells were grown on fibronectin coated 6 well plates. As soon as the well area was completely covered by cells (after approx. 5–6 days), an artificial wound was created, using the tip of a syringe. Cells remained in either Angiopoietin-1 free EBM-2 or in Ang-1 containing medium (250 ng/ml). Incubation time was one hour in every series of experiments. Each series was performed three times. After cell washing with Ang-1 free EBM-2, images of the respective wound areas were taken at 0 and 24 hours. Commercially available human umbilical vein endothelial cells (HUVECs – PCS-100-013, ATTC, Wesel, Germany) were cultured on fibronectin coated 6 well plates in EBM-2. An artifical wound was created as described. Cells were incubated with supernatant EBM-2 from eEOCs that were incubated with Ang-1 for one hour. After incubation, supernatant was removed and cells remained in fresh EBM-2 for one more hour. Supernatant was then used for treatment of HUVECs. Incubation time with supernatant was 3 hours. Images of the respective wound areas were taken at 3 and 27 hours. The experiment was performed at least three times.

### ELISA studies

For all in vitro cell experiments, a commercially available murine ’early outgrowth’ endothelial progenitor cell line was purchased (66110–37 - Celprogen stem cell research and therapeutics, San Pedro, CA, USA). Cells were cultured according to the manufacturer’s protocol. Cell treatment with Ang-1 was performed as described above. For measuring levels of Vascular Endothelial Growth Factor, Insulin-like Growth Factor-1, Interleukin-6, and Transforming Growth Factor-β in the culture medium at 24 hours after cell treatment, commercially available ELISA tests were performed according to the manufacturer’s protocol (R&D Systems, Minneapolis, MN, USA). For measuring tissue levels of IL-6 and TGF-β, frozen kidney samples were thawed and frozen for 3 times in PBS. After the last cycle, tissue homogenates were centrifuged at 5.000 g and supernatants were employed for ELISA studies.

### Analysis of eEOC apoptosis and necrosis

For analyzing the effects of Ang-1 on eEOC apoptosis and necrosis, cultured murine eEOCs were incubated with either medium alone or with Ang-1 containing medium (250 ng/ml). As reported earlier [23], apoptosis/necrosis was induced by treating the cells with TGF-β. Cells were first incubated with TGF-β (TGF-β 5 ng/ml), in some experiments Ang-1 (250 ng/ml) was added after a period of 2 hours, both mediators remained in the culture medium for one more hour. Either untreated or pretreated cells (TGF-β alone, TGF-β/Ang-1 combined) were washed and 24 hours later apoptosis and necrosis were evaluated. For analyzing apoptosis, the percentage of Annexin V + cells was quantified using a commercially available kit (BD, Heidelberg, Deutschland) according to the manufacturer’s protocol. Necrosis was measured by cytometric analysis. Briefly, cells with positive uptake of propidium iodide were defined as necrotic.

### Statistical analysis

The results were expressed as mean ± SEM. The means of two populations were compared by Student’s t test (unpaired with equal variance). Differences between three or more groups were compared by ANOVA one way analysis of variance. Differences were considered significant at p < 0.05.

## Results

### Angiopoietin-1 increases renoprotective competence of eEOCs in iAKI

**Figure 1:**
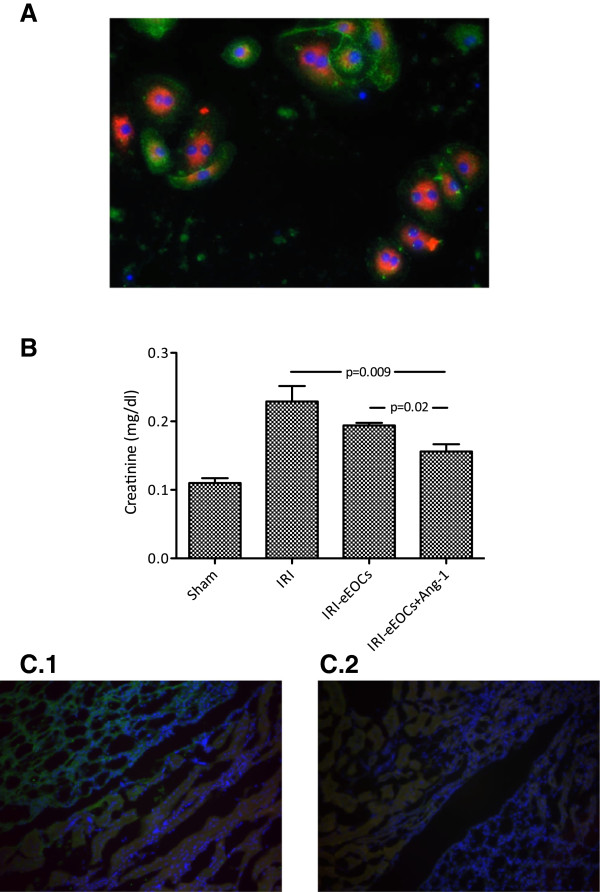
**Cultured murine eEOCs and renal function after cell therapy. A** depicts BS 1^+^/acLDL^+^ cells (eEOCs) (green: BS 1 lectin-FITC, red: acLDL-Dil, blue: nuclei, magnification × 100). Eighty-six ± 3.7% of all cultured cells showed double-positive staining. **B** shows renal function of cell injected animals. Administration of untreated eEOCs did not improve renal function but injection of Ang-1 pretreated cells significantly improved renal function as compared to mice without cell therapy and to mice with administration of untreated eEOCs. **C (C.1** and **C.2)** shows immunofluorescence images from kidneys of cell-injected mice. Since previous investigations showed eEOC accumulation in the medullo-papillary area, our particular interest focused on the borderzone between medulla and papilla. Nevertheless, cell were de facto absent in both groups (untreated cells – **C.1** and Ang-1 pretreated cells – **C.2**) (magnification in C × 40, IRI: ischemia reperfusion injury, Data as mean ± SEM, *: p < 0.05).

After a 40 minutes period of ischemia mice were intravenously injected with 0.5 × 10^6^ Ang-1 pretreated cells. Serum creatinine levels of cell injected animals significantly improved as compared to postischemic mice that did not receive any cells at all or that received untreated cells (0.15 ± 0.01 mg/dl vs. 0.22 ± 0.03 mg/dl or vs. 0.19 ± 0.004 mg/dl, p = 0.009 and p = 0.02 – Figure 1).

### Angiopoietin-1 does neither increase nor decrease postischemic eEOC homing

In order to analyze postischemic cell homing, some experiments required eEOC labelling with cell tracker® solution (CM-Dil). Cell tracker® incubation of eEOCs was exclusively performed for analyzing cell homing since preliminary experiments using Ang-1 pretreated cells showed that renal function can deteriorate after labelling the cells (data not shown). Immunofluorescence analysis showed *de facto* no cell infiltrates in all injected animals as it has been reported in previous studies [5,23,24]. Representative images are shown in Figure 1.

### Angiopoietin-1 stimulates migration of cultured murine eEOCs

**Figure 2:**
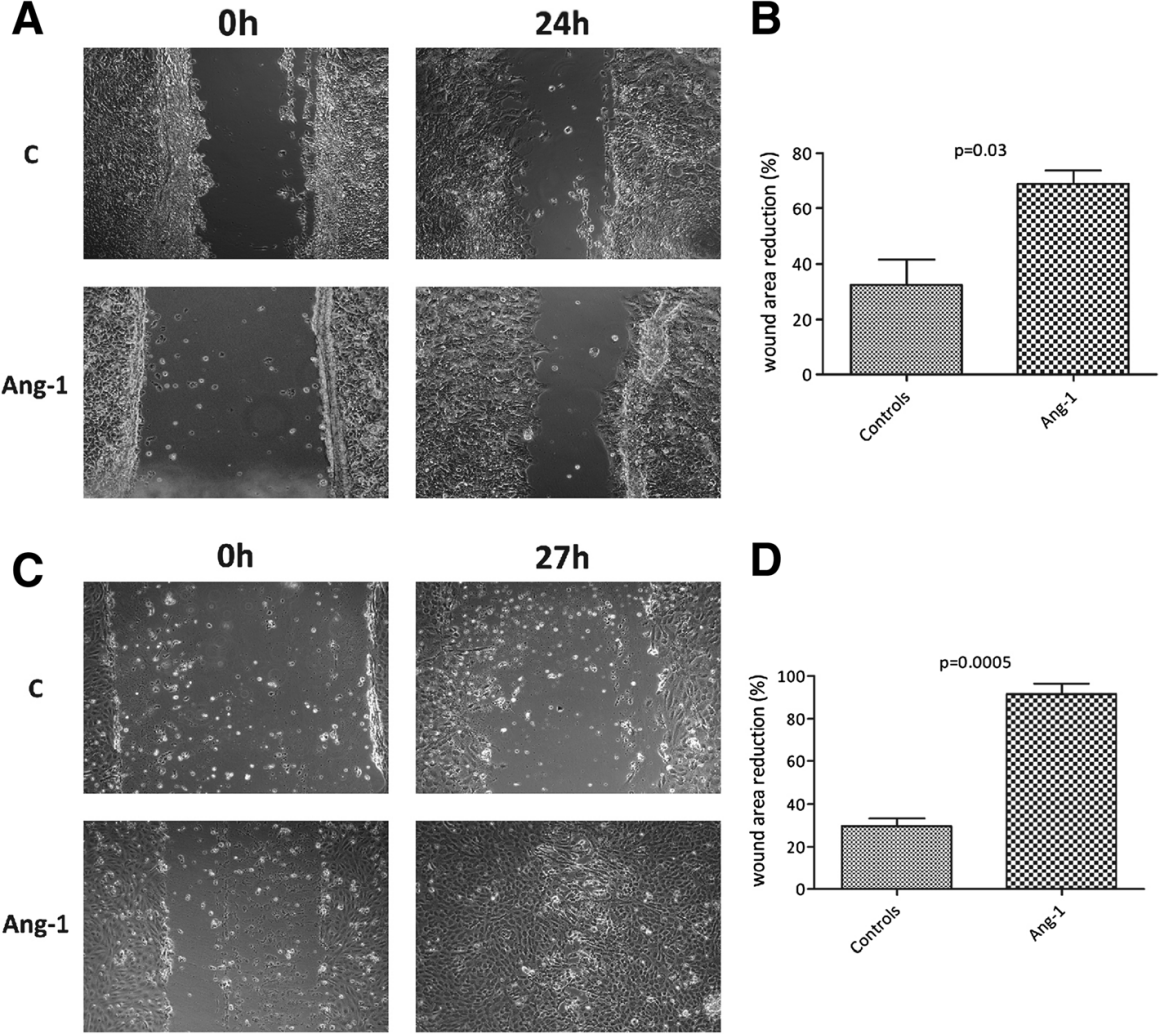
**Effects of Ang-1 on migratory activity of cultured eEOCs (A) and cultured mature endothelial cells (C).** The latter were incubated with medium from Ang-1 treated eEOCs. Ang-1 induced faster direct and indirect wound closing, indicating proendothelial effects of the protein (A: direct wound healing assay with C – Control; **B**: wound area reduction after 24 hours; **C**: indirect wound healing assay with C – Control, **D**: wound area reduction after 27 hours). Number of analysis in each group: 6, Data as mean ± SEM, *: p < 0.05).

We next aimed to determine the migratory activity of cultured eEOCs in the presence of Ang-1. Angiopoietin-1 significantly accelerated wound closing as indicated by smaller wound areas at 24 hours after treatment (69 ± 8% wound closing vs. 32 ± 22% wound closing, p = 0.03, Figure 2).

### Angiopoietin-1 stimulates migration of cultured human endothelial cells in an indirect manner

After artificial wound induction, human umbilical vein endothelial cells were incubated with medium from Ang-1 treated eEOCs. This measure significantly accelerated wound closing (92 ± 8% wound closing vs. 30 ± 6% wound closing, p = 0.0005, Figure 2), indicating that Angiopoietin-1 treatment of eEOCs increases indirect proangiogenic effects of the cells.

### Angiopoietin-1 does not increase eEOC release of proangiogenic or inflammatory mediators

**Figure 3:**
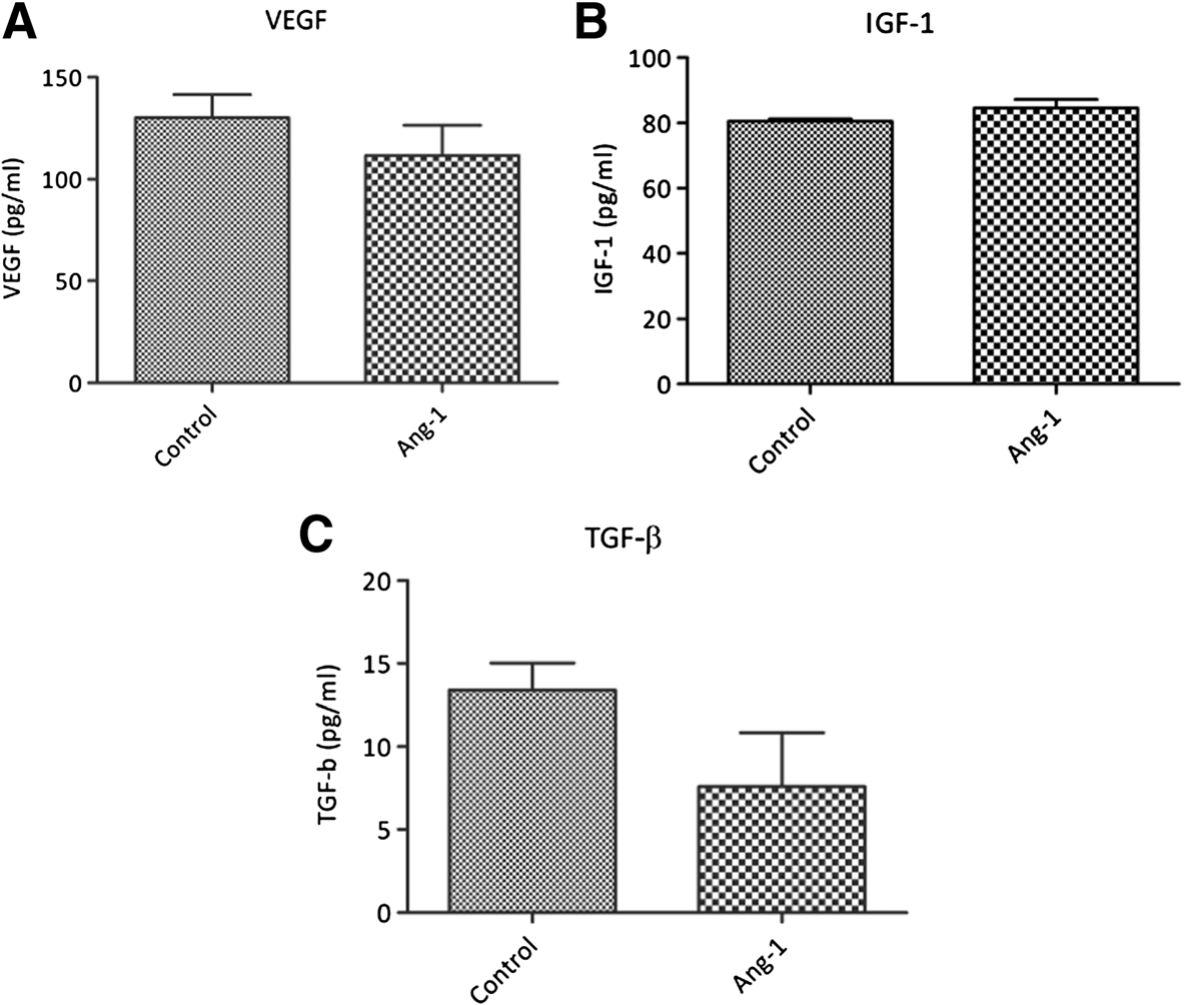
**Analysis of eEOC release of VEGF (A), IGF-1 (B), and TGF-β (C).** There were no differences between untreated and Ang-1 pretreated eEOCs (Data as mean ± SEM).

We next evaluated eEOC production/secretion of different proangiogenic and proinflammatory mediators. Twenty-four hours after a one hour period of Ang-1 treatment, concentrations of vascular endothelial growth factor (VEGF), Insulin-like Growth Factor-1 (IGF-1), Interleukin-6 (IL-6), and Transforming Growth Factor-β (TGF-β) were not different in the supernatants from untreated as compared to Ang-1 treated eEOCs (Figure 3). Results of IL-6 measurements are not part of Figure 3 since the cytokine was almost not detectable at all in supernatant from untreated/treated cells.

### Angiopoietin-1 reduces apoptosis/necrosis of eEOCs

**Figure 4:**
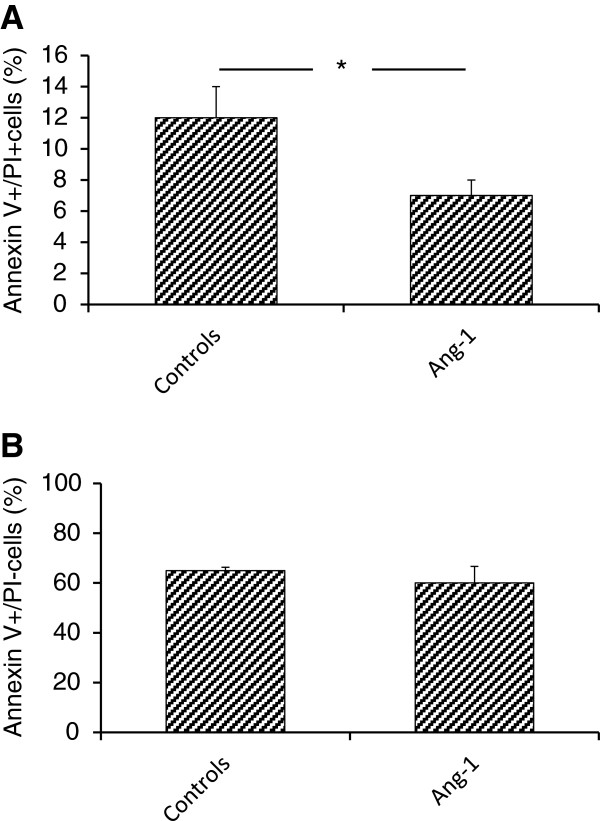
**Apoptosis and necrosis of Ang-1 treated eEOCs.** Percentages of Annexin V^+^/PI^+^ (double-positive) cells were significantly lower in Ang-1 incubated cells as compared to untreated eEOCs **(A)**. **B** shows percentages of Annexin V^+^/PI^-^ cells. Percentages did not differ bewteen the groups which indicated that differences in double-positive cells were the result of increased necrosis rather than apoptosis (Data as mean ± SEM, *: p < 0.05).

Finally, we evaluated whether Angiopoietin-1 modulates TGF-β induced apoptosis/necrosis of cultured murine eEOCs. Twenty-four hours after a one hour period of Angiopoietin-1 treatment, eEOC were analyzed for Annexin V expression and propidiumiodide uptake. Angiopoietin-1 significantly reduced percentages of double-positive cells (6.8 ± 1.1% vs. 11.6 ± 1.9%, p = 0.0004 – Figure 4). In fact both processes of cell damage (apoptosis and necrosis) were reduced in the presence of Ang-1 but necrosis, as represented by uptake of PI was decreased to an even greater extent. Thus, the Annexin V/PI ratio was diminished as well.

## Discussion

Early Endothelial Outgrowth Cells significantly protect mice from acute ischemic renal failure. This has meanwhile been proven in a number of different experimental studies [5,23-26]. In attempting to establish the cells as future therapeutic tool in ischemic AKI, different strategies have been identified, helpful to increase renoprotective effects of eEOCs. In 2010, 8-O-cAMP was identified as eEOC agonist, inducing redistribution of integrin molecules towards the plasma membrane of the cells thereby enhancing postischemic cell homing [24]. The hormone melatonin improved antiischemic actions of eEOCs in AKI as well [23]. Recently, the protein BMP-5 was identified as another eEOC agonist in this situation [27]. Thus, Early Endothelial Outgrowth Cells increasingly become a therapeutic option in ischemic AKI.

Aim of the current study was to analyze consequences of Angiopoietin-1 (Ang-1) treatment of eEOCs in the context of eEOC-mediated postischemic kidney repair. Pretreatment of the cells resulted in significant lower concentrations of serum creatinine in cell-injected mice, which was accompanied by higher resistance of cultured eEOCs against TGF-β induced apoptosis/necrosis and by increased cell migration. In addition, supernatant from Ang-1 incubated eEOCs markedly enhanced migratory activity of cultured mature endothelial cells. Postischemic cell homing or production/secretion of proangiogenic mediators remained unaffected by Ang-1. The latter findings are somehow surprising, regarding the fact that eEOCs predominantely act through secreted signals. The cells home into the interstitial space of postischemic tissues where they release proangiogenic mediators in a paracrinic manner [28]. Miscellaneous substances have been identified in the past, including VEGF, HGF, IGF-1 [28,29], matrix metalloproteinase 9, IL-8, and others [30,31]. Although VEGF plays a fundamental role in orchestrating protective effects of the cells [23,28], we did not find increased cellular secretion of VEGF (and HGF). An alternative mechanism of EPC(eEOC)-mediated vasoprotection has lately been introduced by the group of Goligorsky [32]. Yasuda and colleagues showed direct interaction between EPCs and mature endothelial cells by the generation of so-called nanotubes, cell membrane-build structures which allow direct transfer of lysosome constituents and other molecules from one cell to the other [32]. This study indicated the potential relevance of direct cell-cell communication, in addition to indirect paracrinic EPC effects. However, our investigation did [5,23,24] not reveal any relevant cell incorporation into the postischemic endothelium or any accumulation of eEOCs in close proxomity to endothelial cells. This is by the way in line with the currently favored concept of eEOC-mediated vasorepair [26,30]. The virtual absence of eEOCs in the postischemic kidney at 48 hours has been reported in previous eEOC/AKI-related studies [23,33] and comparable data has been documented for mesenchymal stem cells as well [34]. So far, significant and increased postischemic eEOC homing has only been observed in one of our studies. The substance 8-O-cAMP induced redistribution of β1-integrin molecules towards the plasma membrane of eEOCs resulting in increased transvascular cell migration into the interstitial space [24]. Nevertheless, our analyses of postischemic kidneys were performed 48 hours after ischemia. Severe ischemia-related endothelial dysfunction typically occurs within the first 24 hours and the absence of injecetd cells at the end of day two only supports the concept of (transient) paracrinic cell actions of eEOCs [35]. Angiopoietin-1 plays a crucial role in maintaining vascular homeostasis under physiological conditions and this study undoubtedly proves an eEOC-agonistic role of the protein in iAKi although it still remains to be elucidated which excact mechanisms promote eEOC renoprotection in vivo. At this point, several endogenous [25] and exogenous agonists of eEOCs have been identified (8-O-cAMP, Melatonin, BMP-5, Ang-1 [23,24,27]). A first study on therapeutic administration of eEOCs in human AKI is being planned at the moment. Thus, exogenous measures, helpful to improve renoprotective competence of the cells in ischemic AKI are needed. Another important question with regard to an EPC-based therapy of ischemic AKI is related to the mid- to long-term outcome of cell treated animals/patients. It has to be analyzed whether EPCs diminish postischemic vascular rarefication/interstitial fibrosis, two events which substantially worsen prognosis of patients in the long-term.

In summary, Angiopoietin-1 has been identified as potent agonist of syngeneic murine eEOCs in ischemic AKI. The protein increases renoprotective effects of the cells in vivo and activates cultured cells on different levels. Angiopoietin-1 is therefore a promising tool for stimulating eEOC-mediated renoprotection in ischemic AKI.

## Conclusions

In conclusion, Angiopoietin-1 has been identified as new agonist of murine eEOCs in ischemic AKI. The substance does not increase postischemic cellular homing into the kidney but significantly promotes cellular activity as reflected by increased survival and migration. In addition, Ang-1 treated eEOCs stimulate competence of mature endothelial cells through secreted signals. Thus, the protein will potentially serve as candidate for priming eEOCs in an eEOC-based therapeutic regimen of AKI.

## Abbreviations

Ang-1: Angiopoietin-1; eEOCs: Early Endothelial Outgrowth Cells; EPCs: Endothelial Progenitor Cells; iAKI: Ischemic acute kidney injury.

## Competing interests

The authors declare that they have no competing interests.

## Authors’ contributions

DP: study design, analysis of data and drafting of the manuscript. JR: surgery of mice. NI: surgery of mice. RB: surgery of mice and tissue sections. KS: assistance with surgery, tissue sections, morphology. EH: in vitro analyses. SP: in vitro analyses. GAM: correction of manuscript. All authors read and approved the final manuscript.

## Pre-publication history

The pre-publication history for this paper can be accessed here:

http://www.biomedcentral.com/1471-2369/14/227/prepub
